# microRNA-19a protects osteoblasts from dexamethasone via targeting TSC1

**DOI:** 10.18632/oncotarget.23326

**Published:** 2017-12-15

**Authors:** Gang Liu, Feng-Li Chen, Feng Ji, Hao-Dong Fei, Yue Xie, Shou-Guo Wang

**Affiliations:** ^1^ Department of Orthopedics, Huai’an First People's Hospital, Nanjing Medical University, Huai’an, China; ^2^ Clinical Laboratory, Huai’an First People's Hospital, Nanjing Medical University, Huai’an, China

**Keywords:** dexamethasone, osteoblasts, microRNA-19a, tuberous sclerosis complex 1 (TSC1), mTOR complex 1 (mTORC1)

## Abstract

Activation of mTOR complex 1 (mTORC1) could protect human osteoblasts from dexamethasone. Tuberous sclerosis complex 1 (TSC1) is mTORC1 upstream inhibitory protein. We demonstrate here that microRNA-19a (“miR-19a”, -3p) targets the 3' untranslated regions of *TSC1 mRNA*. Expression of miR-19a downregulated TSC1 in OB-6 osteoblastic cells and primary human osteoblasts. miR-19a activated mTORC1 and protected human osteoblasts from dexamethasone. mTORC1 inhibition, by RAD001 or Raptor shRNA, almost completely abolished miR-19a-induced osteoblast cytoprotection against dexamethasone. Knockdown of TSC1 by targeted shRNA similarly induced mTORC1 activation and protected osteoblasts. Moreover, miR-19a activated mTORC1-dependent NF-E2-related factor 2 (Nrf2) signaling and inhibited dexamethasone-induced reactive oxygen species production in osteoblasts. Together, miR-19a protects human osteoblasts from dexamethasone possibly via targeting TSC1-mTORC1 signaling.

## INTRODUCTION

Dexamethasone (Dex) shall induce direct injuries to human osteoblasts [1–3]. In the bones of Dex-taking patients, reduced number of osteoblasts and increased osteoblast cell apoptosis are often detected [4, 5]. Our group [6–12] and others [13–15] have been adding Dex to the cultured human osteoblasts *in vitro*, serving as a cellular model of glucocorticoid-induced osteoblast injuries.

Tuberous sclerosis complex 1 (TSC1)-TSC2 protein complex is an upstream inhibitor of mTOR complex 1 (mTORC1) [16, 17]. Inhibition, silence, mutation or disruption of TSC1-TSC2 protein complex would induce mTORC1 activation, leading to phosphorylations of its downstream substrate proteins, including p70S6 kinase (p70S6K) and eukaryotic initiation factor 4E-binding protein 1 (4E-BP1) [18–20].

mTORC1 activation could promote cell survival [16, 17]. Recent studies have implied that activation of mTORC1 could also protect osteoblasts from Dex [6, 21]. The current study aims to induce mTORC1 activation by silencing TSC1 using microRNA strategy. miRNA can suppress expression of targeted-gene via binding to 3′-untranslated region (UTR) [22–24]. We show that microRNA-19a (“miR-19a”) directly targets and downregulates TSC1, causing mTORC1 activation in human osteoblasts. Importantly, miR-19a expression protects human osteoblasts from Dex.

## RESULTS

### Forced-expression of miR-19a downregulates TSC1 in human osteoblasts

**Figure 1:**
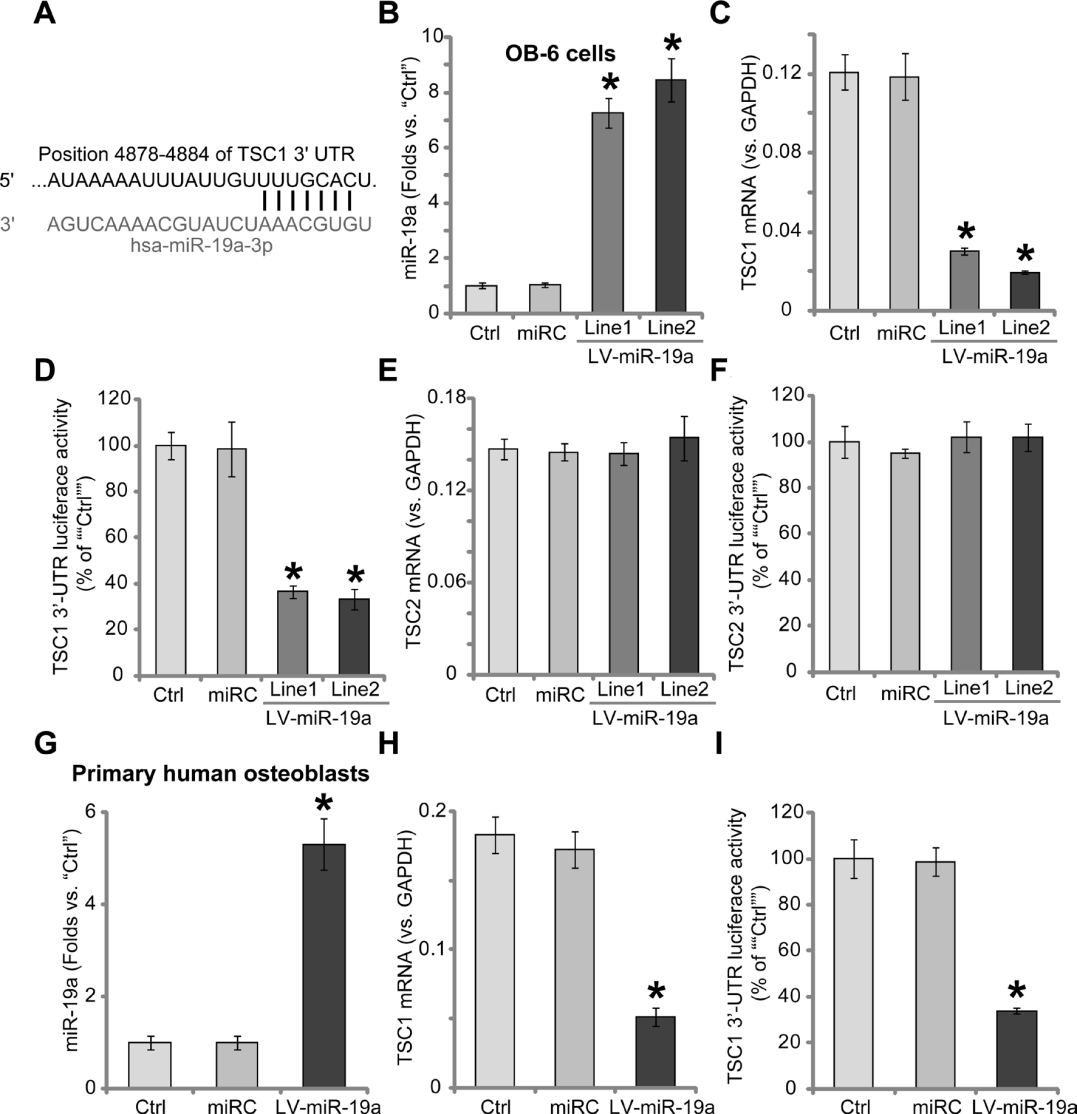
Forced-expression of miR-19a downregulates TSC1 in human osteoblasts microRNA-19a-3p (“miR-19a”) putatively targets the 3′-UTR of human *TSC1 mRNA* (**A**). Stable OB-6 osteoblastic cells, expressing the lentiviral miR-19a expression vector (“LV-miR-19a”, two lines, “Line1/2”), non-sense scramble control microRNA (“miRC”), or the parental control OB-6 cells (“Ctrl”), were subjected to quantitative real-time PCR (“qRT-PCR”) assay of miR-19a (“-3p”) and *TSC1/2 mRNA* expressions (**B**, **C** and **E**); *TSC1/2 mRNA* 3′-UTR luciferase activity assay was also tested (**D** and **F**). Primary human osteoblasts were infected with the LV-miR-19a or miRC for 48 hours, miR-19a (“-3p”) expression (**G**), *TSC1 mRNA* level (**H**) and *TSC1 mRNA* 3′-UTR luciferase activity (**I**) were tested similarly. Data were expressed as mean ± SD (*n* = 5). ^*^p<0.05 *vs.* “Ctrl”. Experiments in this figure were repeated five times, and similar results were obtained.

Results in Figure 1A showed that miR-19a (“-3p”) targets the 3′-untranslated region (UTR) of human TSC1 (at position 4878–4884). A lentiviral miR-19a expression vector (“LV-miR-19a”) was established, which was introduced to OB-6 human osteoblastic cells. Cells were subjected to puromycin selection. Two lines of stable OB-6 cells with LV-miR-19a were established, which were named as “LV-miR-19a (Line-1)” and “LV-miR-19a (Line-2)”. The quantitative real-time PCR (“qRT-PCR”) assay results in Figure 1B demonstrated that miR-19a (“-3p”) expression level was dramatically increased in the two lines of OB-6 cells. (Figure 1B). Remarkably, *TSC1 mRNA* expression was decreased in the two lines of LV-miR-19a OB-6 cells (Figure 1C). Meanwhile, the *TSC1 mRNA*'s 3′ untranslated regions (3′-UTR) luciferase activity was also inhibited after miR-19a expressing (Figure 1D), indicating that miR-19a directly targets *TSC1 mRNA*. *mRNA* expression (Figure 1E) and 3′-UTR luciferase activity (Figure 1F) of TSC2, a protein that forms complex with TSC1, were unchanged by LV-miR-19a.

We also repeated the above experiments in the primary human osteoblasts. As demonstrated, forced-expression of miR-19a by LV-miR-19a (Figure 1G) led to significant *TSC1 mRNA* downregulation (Figure 1H). Meanwhile, *TSC1 mRNA*'s 3′-UTR luciferase activity was also decreased by LV-miR-19a (Figure 1I). The non-sense scramble control microRNA (“miRC”) didn't change miR-19a nor TSC1/2 expressions in the osteoblasts (Figure 1B–1I). Collectively, these results indicate that forced-expression of miR-19a induces TSC1 downregulation in human osteoblasts.

### miR-19a downregulates TSC1/2 and activates mTORC1 in human osteoblasts

**Figure 2:**
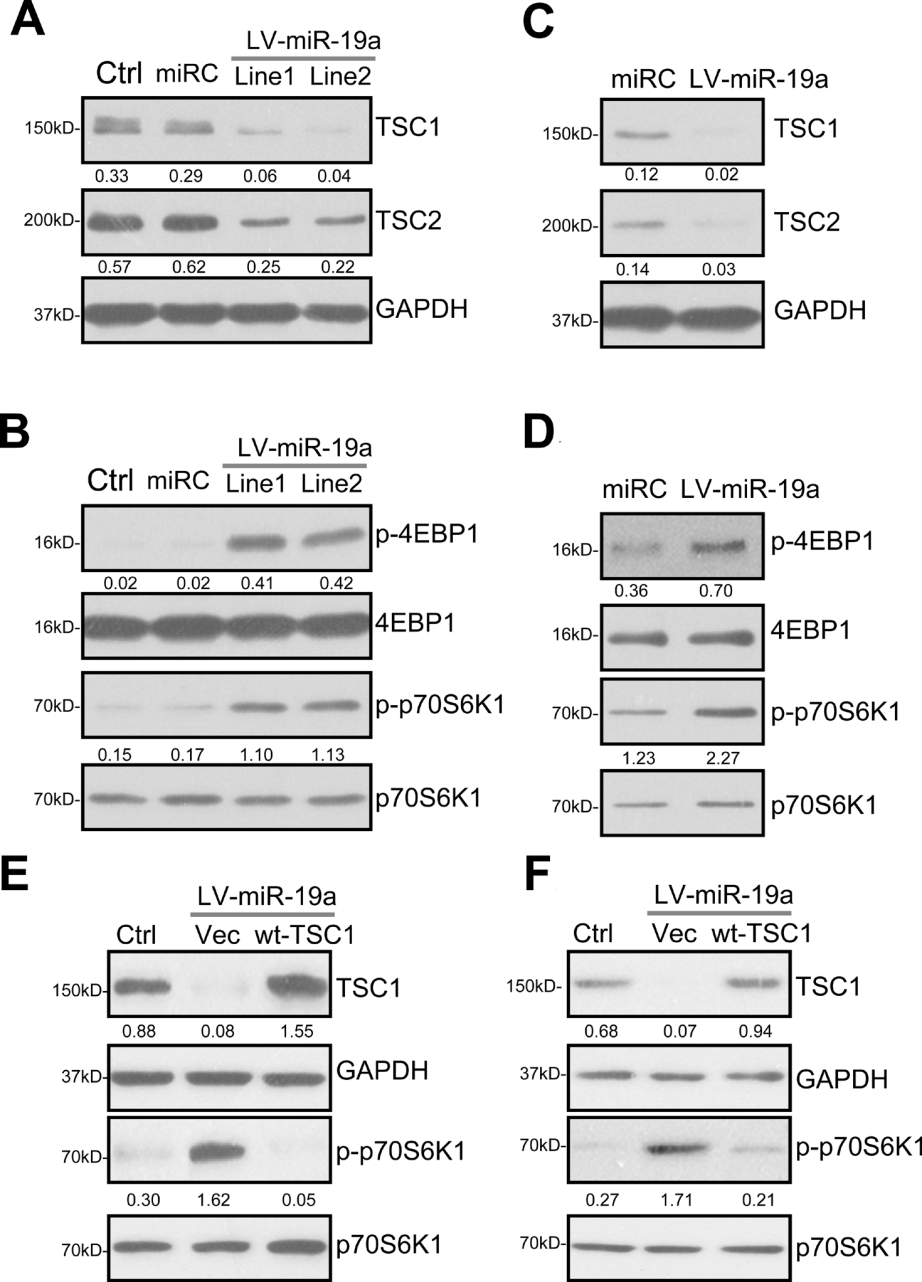
miR-19a downregulates TSC1/2 and activates mTORC1 in human osteoblasts Stable OB-6 cells, expressing the lentiviral miR-19a expression vector (“LV-miR-19a”, two lines, “Line1/2”), non-sense scramble control microRNA (“miRC”), or the parental control OB-6 cells (“Ctrl”), were subjected to Western blotting assay of listed proteins (**A** and **B**). Primary human osteoblasts were infected with LV-miR-19a or miRC for 48 hours, expressions of listed proteins were shown (**C** and **D**). Stable OB-6 cells with LV-miR-19a (“Line1”) (**E**), as well as the primary human osteoblasts with LV-miR-19a (**F**), were further transfected with TSC1 cDNA construct (“wt-TSC1”, 0.20 μg/mL, for 48 hours) or the empty vector (pSuper-puro-EGFP, “Vec”), total cell lysates were subjected to Western blotting assay of listed proteins. The indicated protein band was quantified (total gray), and its value was normalized to the loading control. Experiments in this figure were repeated four times, and similar results were obtained.

In the unstimulated condition, TSC1 forms a complex with TSC2, inhibiting mTORC1 [16, 17, 25–27]. Inhibition of this complex, *i.e.* by AKT-dependent of phosphorylation of TSC2, will lead to downstream mTORC1 activation [16, 17, 25–27]. Importantly, TSC1 acts as a stabilizer of TSC2 by protecting it from ubiquitin-dependent degradation [28]. TSC1 silence will lead to TSC2 ubiquitination and degradation [16, 17, 25–27]. The results in Figure 1 demonstrated that expression of miR-19a by LV-miR-19a caused *TSC1 mRNA* depletion. Western blotting assay results in Figure 2A showed that TSC1 protein expression was also decreased in two lines of LV-miR-19a-expressing OB-6 cells. Importantly, TSC2 protein level was also reduced by LV-miR-19a (Figure 2B). Considering that *TSC2 mRNA* was unchanged in LV-miR-19a OB-6 cells (Figure 1E), TSC2 downregulation by miR-19a should be due to the disruption of the TSC1-TSC2 complex. Remarkably, as shown in Figure 2B, phosphorylations of two key mTORC1 substrates, including 4EBP1 and p70S6K1 [19, 20, 29, 30], were significantly increased in miR-19a-expressing OB-6 cells, suggesting mTORC1 activation.

We repeated the experiments in the primary human osteoblasts. As demonstrated, introduction of LV-miR-19a also induced protein downregulation of TSC1 and TSC2 in the primary osteoblasts (Figure 2C). mTORC1 activation (p-4EBP1/p-S6K1) was significantly increase (Figure 2D). miRC, as expected, failed to change TSC1/2 expression nor mTORC1 activation (Figure 2A–2D).

To further confirm that TSC1 downregulation is the direct cause of mTORC1 activation in human osteoblasts, a TSC1-expression construct (see Method) was introduced to miR-19a-expressing OB-6 cells. The construct restored TSC1 expression (Figure 2E). Importantly, exogenous TSC1 expression in OB-6 cells almost completely blocked miR-19a-induced mTORC1 activation (tested by p-S6K1, Figure 2E). In miR-19a-expressing primary human osteoblasts, the TSC1 construct similarly restored TSC1 expression and abolished mTORC1 activation (Figure 2F). As expected, the TSC1 construct didn't affect miR-19a expression by LV-miR-19a in the osteoblasts (Data not shown). These results imply that TSC1 silence should be the direct and primary cause of mTORC1 activation by miR-19a in human osteoblasts.

### miR-19a protects human osteoblasts from Dex

**Figure 3:**
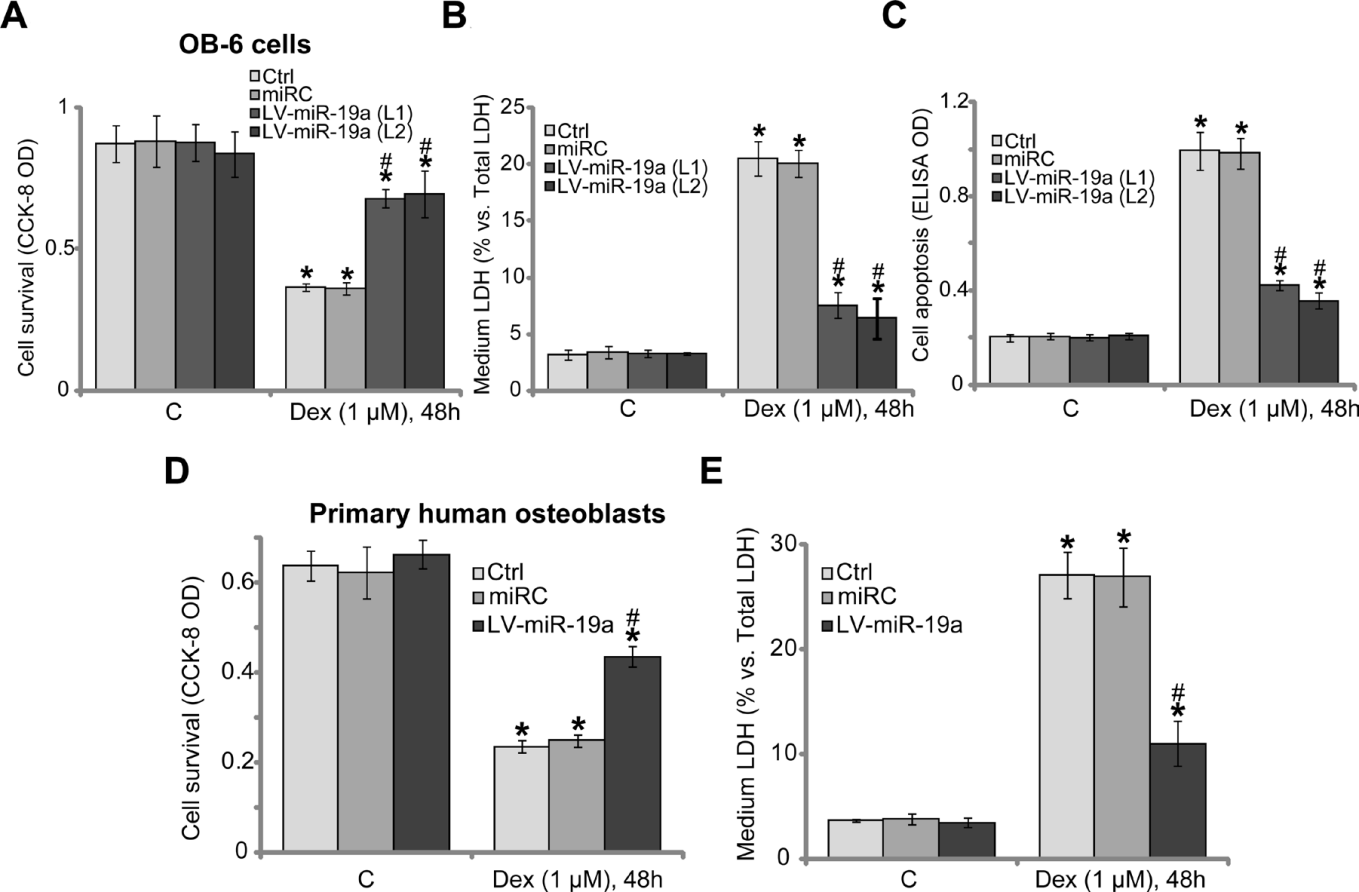
miR-19a protects human osteoblasts from Dex OB-6 osteoblastic cells (**A**–**C**) or the primary human osteoblasts (**D**–**E**), with miR-19a expression vector (“LV-miR-19a”) or the non-sense scramble control microRNA (“miRC”), as well as the parental control cells (“Ctrl”), were treated with/out Dex (1 μM) for 48 hours, cell viability (CCK-8 assay, A and D), cell death (LDH release assay, B and E) and apoptosis (Histone DNA ELISA assay, C) were examined. Data were expressed as mean ± SD (*n* = 5). “C” stands for untreated control group. ^*^p < 0.05 *vs.* “C”. ^#^*p* < 0.05 *vs.* Dex treatment of “miRC” cells. Experiments in this figure were repeated three times, and similar results were obtained.

Studies [6, 21] have shown that activation of mTORC1 could protect human osteoblasts from Dex, we therefore tested whether miR-19a expression could also exert similar functions. In line with our previous findings [6–11], treatment with Dex (1 μM, 48 hours) in the control OB-6 cells induced potent viability reduction [Cell Counting Kit-8 (CCK-8) optic density (OD) decrease, Figure 3A), cell death [lactate dehydrogenase (LDH) release, Figure 3B] and apoptosis (Histone DNA ELISA OD increase, Figure 3C). Remarkably, Dex-induced cytotoxicity was dramatically attenuated in the two lines of OB-6 cells with LV-miR-19a (Figure 3A–3C). Thus, miR-19a expression indeed protected OB-6 cells from Dex. The similar results were also observed in the primary human osteoblasts, where LV-miR-19a attenuated Dex-induced cell viability reduction (Figure 3D) and cell death (Figure 3E). It should be noted that non-sense scramble control microRNA (“miRC”) didn't inhibit Dex-induced cytotoxicity in human osteoblasts.

### mTORC1 activation is required for miR-19a-mediated osteoblast cytoprotection against Dex

**Figure 4:**
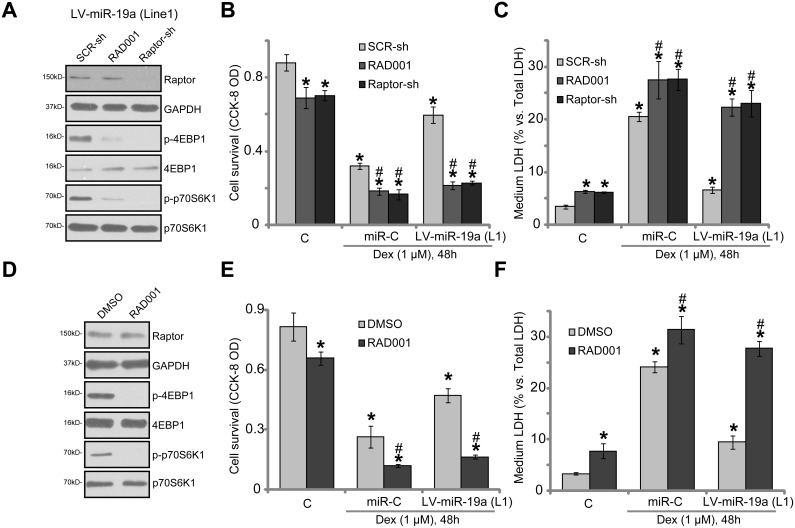
mTORC1 activation is required for miR-19a-mediated osteoblast cytoprotection against Dex OB-6 cells, expressing the lentiviral miR-19a expression vector (“LV-miR-19a”, Line1) or the non-sense scramble control microRNA (“miRC”), were further infected with lentiviral scramble control shRNA (“SCR-sh”) or Raptor shRNA (“Raptor-sh”) for 24 hours, cells were treated with/out Dex (1 μM), or plus RAD001 (200 nM); Expressions of listed proteins were shown (**A**, 1 hour after Dex treatment); Cell survival (**B**) and death (**C**) were tested 48 hours after Dex treatment. Primary human osteoblasts were infected with the LV-miR-19a or miRC for 48 hours, cells were treated with/out Dex (1 μM), or plus RAD001 (200 nM); Expressions of listed proteins were shown (**D**, 1 hour after Dex treatment); Cell survival (**E**) and death (**F**) were tested 48 hours after Dex treatment. Data were expressed as mean ± SD (*n* = 5). “C” stands for untreated control group. ^*^p < 0.05 *vs.* “C”.^#^*p* < 0.05 *vs.* Dex treatment of “SCR-sh” cells (B and C) or “DMSO” cells (E and F). Experiments in this figure were repeated three times, and similar results were obtained.

In order to prove that mTORC1 activation is required for miR-19a-mediated osteoblast cytoprotection, pharmacological and genetic methods were employed to block mTORC1 activation. RAD001, a well-known specific mTORC1 inhibitor, was applied [31, 32]. Since long-term RAD001 treatment might also inhibit mTOR complex 2 (mTORC2) activation [33], shRNA method was utilized to knockdown Raptor in OB-6 cells. The latter (Raptor) is key component of mTORC1 [34–36]. Notably, RAD001 or Raptor shRNA almost completely blocked mTORC1 activation (p-S6K1/p-4EBP1) in OB-6 cells with LV-miR-19a (“Line1”, Figure 4A). The applied Raptor shRNA potently silenced Raptor in OB-6 cells (Figure 4A). Dex-induced cytotoxicity in OB-6 cells was intensified by RAD001 and Raptor shRNA, leading to increased viability reduction (Figure 4B) and apoptosis (Figure 4C). Thus, mTORC1 activation was cytoprotective in Dex-treated OB-6 cells. Remarkably, miR-19a-induced osteoblast cytoprotection against Dex was almost completely nullified by RAD001 or Raptor shRNA (Figure 4B and 4C). Similarly in the primary human osteoblasts, mTORC1 blockage by RAD001 (Figure 4D) intensified Dex-induced injuries and almost reversed miR-19a-induced anti-Dex actions (Figure 4E and 4F). These results suggest that mTORC1 activation is required for miR-19a-induced osteoblast cytoprotection against Dex.

### TSC1 is the primary target of miR-19a in mediating its cytoprotective activity in osteoblasts

**Figure 5:**
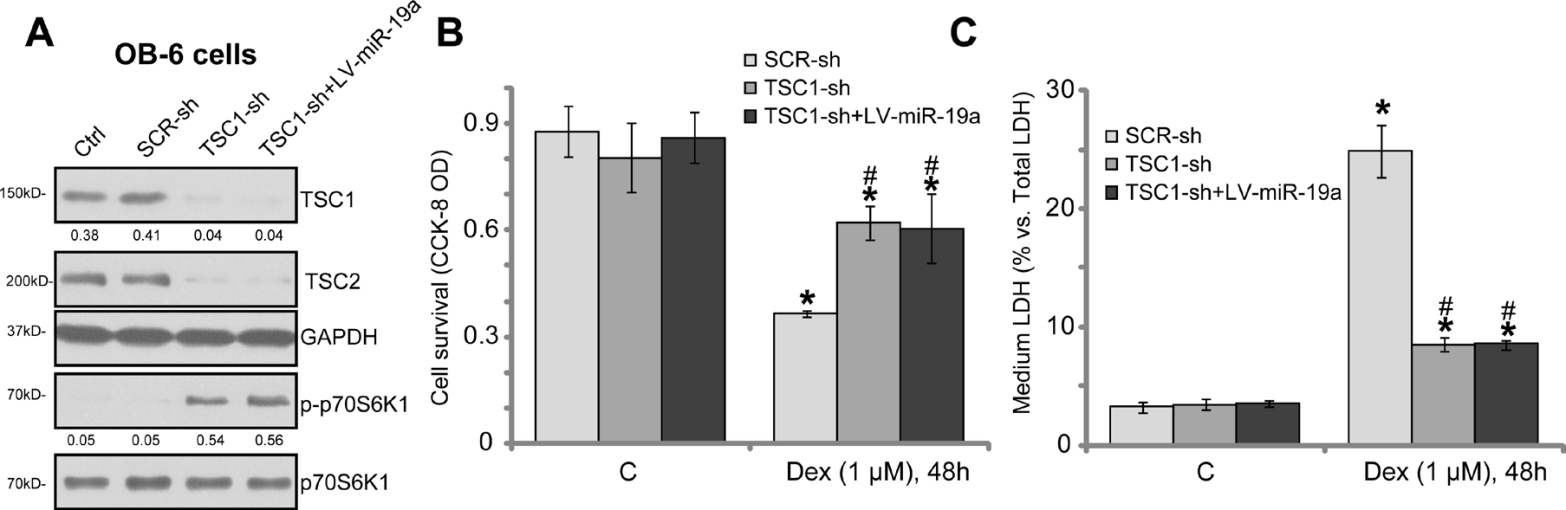
TSC1 is the primary target of miR-19a in mediating its cytoprotective activity in osteoblasts The OB-6 osteoblastic cells were infected with lentiviral scramble control shRNA (“SCR-sh”), TSC1 shRNA (“TSC1-sh”) or plus lentiviral miR-19a expression vector (“+LV-miR-19a”), cells were further selected by puromycin, expressions of listed proteins were shown (**A**). The above-mentioned cells were also treated with/out Dex (1 μM) for 48 hours, cell survival (**B**) and death (**C**) were tested. The indicated protein band wan quantified, and its value was normalized to the loading control (A). Data were expressed as mean ± SD (*n* = 5). “Ctrl” stands for parental control cells. “C” stands for untreated control group. ^*^p < 0.05 *vs.* “C”.^#^*p* < 0.05 *vs.* Dex treatment of “SCR-sh” cells. Experiments in this figure were repeated three times, and similar results were obtained.

If TSC1 is the primary target protein of miR-19a, TSC1 knockdown shall also protect osteoblasts from Dex. To test this hypothesis, shRNA method was again utilized to knockdown TSC1. As demonstrated, the applied lentiviral TSC1 shRNA (purchased from Santa Cruz Biotech) potently downregulated TSC1 in OB-6 cells (Figure 5A). TSC2 protein level was also downregulated (Figure 5A). Correspondingly, mTORC1 activation, or p-S6K1, was boosted (Figure 5A). In the TSC1-shRNA-expressing OB-6 cells, introduction of LV-miR-19a failed to further change TSC1 expression and mTOR activation (Figure 5A). Importantly, OB-6 cells with TSC1 shRNA were also protected from Dex, showing reduced viability reduction (Figure 5B) and cell death (Figure 5C). Intriguingly, expression of miR-19a (by LV-miR-19a) in the TSC1 shRNA-expressing OB-6 cells was unable to further protect cells from Dex (Figure 5B and 5C). These results together indicate that TSC1 is the primary target of miR-19a in mediating its osteoblast cytoprotection.

### miR-19a activates Nrf2 signaling in human osteoblasts

**Figure 6:**
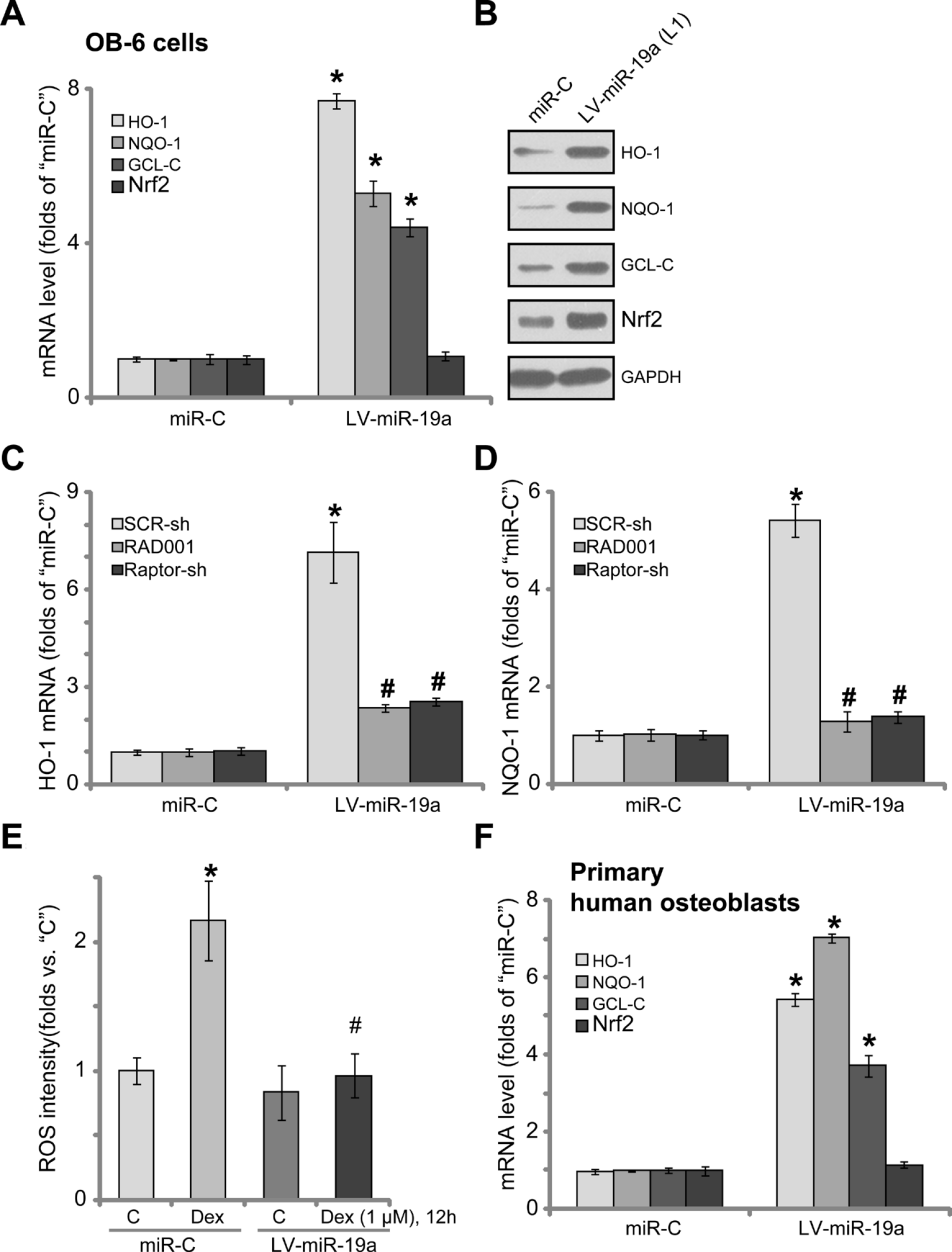
miR-19a activates Nrf2 signaling in human osteoblasts OB-6 osteoblastic cells (**A** and **B**) or primary human osteoblasts (F), with the lentiviral miR-19a expression vector (“LV-miR-19a”) or non-sense scramble control microRNA (“miRC”), were subjected to qRT-PCR assay (A and **F**) and Western blotting assay (B) of listed Nrf2 pathway genes; The OB-6 cells were also treated with/out Dex (1 μM) for 12 hours, relative ROS intensity was analyzed by the DCFH-DA fluorescent dye assay (E). OB-6 cells, with “LV-miR-19a” or “miRC”, were further infected with lentiviral scramble control shRNA (“SCR-sh”) or Raptor shRNA (“Raptor-sh”) for 24 hours, or plus RAD001 (200 nM) co-treatment, qRT-PCR assay was applied to test HO-1 mRNA (C) and NQO-1 mRNA (D); Data were expressed as mean ± SD (*n* = 5). “C” stands for untreated control group. ^*^p < 0.05 *vs.* “miRC”. ^#^*p* < 0.05 *vs.* “SCR-sh” cells (**C** and **D**). ^#^*p* < 0.05 *vs.* Dex treatment of “miRC” cells (**E**). Experiments in this figure were repeated three times, and similar results were obtained.

It has been previously shown that Dex induces reactive oxygen species (ROS) production, which is required for subsequent osteoblast cell death and apoptosis [7, 11, 13, 37]. Activation of mTORC1 could exert anti-oxidant activity via activating downstream NF-E2-related factor 2 (Nrf2) [38, 39]. We thus tested the potential effect of miR-19a on Nrf2 signaling activation. The qRT-PCR assay results in Figure 6A confirmed that expression of miR-19a by LV-miR-19a significantly increased *mRNA* expressions of Nrf2-regulated genes, including *heme oxygenase 1 (HO-1)*, *NAD(P)H quinone oxidoreductase 1 (NQO-1)* and γ-*glutamylcysteine synthetase catalytic subunit (GCL-C)*. HO-1, NQO-1 and GCL-C protein expressions were also upregulated in LV-miR-19a OB-6 cells (Figure 6B). *Nrf2 mRNA* level was unchanged by LV-miR-19a (Figure 6A), yet its protein level was significantly increased (Figure 6B). These results suggest that miR-19a expression induced Nrf2 protein stabilization, which is necessary for its activation [40, 41]. Notably, inhibition of mTORC1, by RAD001 or Raptor shRNA, almost completely blocked LV-miR-19a-induced *mRNA* expression of Nrf2-regulated genes, including *HO-1* (Figure 6C) and *NQO-1* (Figure 6D), indicating that mTORC1 activation is required for miR-19a-mediated Nrf2 activation in osteoblasts. Importantly, results showed that Dex-induced ROS production was largely attenuated following miR-19a expression in OB-6 cells (Figure 6E). Thus, miR-19a expression apparently activated mTORC1-dependent Nrf2 signaling and inhibited Dex-induced oxidative stresses in OB-6 cells. In the primary human osteoblasts, introduction of LV-miR-19a also induced *mRNA* expressions of *HO-1*, *NQO-1* and *GCL-C* (Figure 6F).

## DISCUSSION

In the current study, we propose that miR-19a is a TSC1-targeting miRNA in human osteoblasts. Expression of miR-19a downregulated TSC1 and activated mTORC1 signaling in both OB-6 osteoblastic cells and primary human osteoblasts. Significantly, miR-19a protected human osteoblasts from Dex, and activation of mTORC1 is required for the process. mTORC1 inhibition, by RAD001 or Raptor shRNA, almost completely abolished miR-19a-induced osteoblast cytoprotection. Similarly, TSC1 shRNA activated mTORC1 and protected osteoblasts from Dex. Notably, miR-19a was in valid in TSC1-silenced OB-6 cells. These results imply that TSC1 should be the primary and direct target protein of miR-19a in mediating its cytoprotective activity in osteoblasts. Indeed, we show that *TSC1 mRNA*'s 3′-UTR luciferase assay was also largely attenuated after miR-19a expression.

Our group [7–9, 11] and others have demonstrated that Dex induces reactive oxygen species (ROS) production, which mediates following osteoblast cell apoptosis [9, 42]. Reversely, inhibition of oxidative stress protects osteoblasts from Dex [42–44]. A novel AMP-activated protein kinase (AMPK) activator compound 13 protected osteoblasts from Dex via inhibiting ROS production [9]. Similarly, activation of EGFR-Akt-Nrf2 signaling by icariside II inhibited Dex-induced oxidative stress and osteoblast cell apoptosis [11]. Additionally, SC79-induced osteoblast cytoprotection against Dex was also due to ROS scavenging [37]. In the current study, miR-19a expression inhibited Dex-induced ROS production in osteoblasts. This could explain the superior cytoprotective activity of miR-19a against Dex in human osteoblasts.

The transcription factor Nrf2 signaling is one key cellular defense mechanism against oxidative stress [40, 45]. Recent studies have proposed that mTORC1 could be a upstream signaling for Nrf2 activation [38, 39]. Activated mTORC1 could induce Nrf2 phosphorylation at Ser-40, causing its departure from Keap1. This will lead to Nrf2 stabilization, its nuclear translocation and activation [38, 39]. Here, expression of miR-19a induced Nrf2 stabilization and expression of Nrf2-regulated genes (*HO-1*, *NQO-1* and *GCL-C*). Inhibition of mTORC1, by RAD001 or Raptor shRNA, almost completely blocked miR-19a-induced mRNA expression of Nrf2-regulated genes. These results indicate that miR-19a-induced mTORC1 activation could possibly activate Nrf2 signaling, which likely inhibits Dex-induced oxidative stress. The detailed mechanism may warrant further characterizations.

## CONCLUSIONS

Taken together, miR-19a protects human osteoblasts from Dex possibly via targeting TSC1-mTORC1 signaling.

## METHODS

### Chemicals and reagents

Dexamethasone (Dex), puromycin and RAD001 were provided by Sigma Aldrich (Nantong, China). Cell culture reagents were purchased from Gibco Co. (Nantong, China). All the antibodies utilized in this study were obtained from Cell Signaling Technology (Nanjing, China). Lipofectamine 2000 reagent was provided by Invitrogen (Suzhou, China). All the primers were from Genepharm (Shanghai, China) unless otherwise mentioned.

### OB-6 cell culture

The adherent OB-6 osteoblastic cells were seeded at a density of 5000 cells/cm^2^ and were cultured in α-minimal essential medium (MEM) with 10% fetal bovine serum (FBS), plus 1% each of penicillin, streptomycin, and glutamine.

### Culture of primary human osteoblasts

The redundant trabecular bone fragments were obtained from the written-informed consent healthy donors at Huai’an First People's Hospital (Huai’an, Jiangsu, China). The trabecular bone fragments were minced and washed 2–3 times with warm PBS, which were then digested using DNase and collagenase type II. After 2 h digestion at 37°C, bone debris was separated by using a 70-μm nylon mesh (Falcon). Cells were precipitated by centrifugation, washed several times to remove excess collagenase and DNase enzymes. The primary osteoblasts were then resuspended in α-MEM supplemented with 10% FBS, which were plated at 2 × 10^4^ cells/cm^2^. After confluence, cells were trypsinized, resuspended, and seeded at 1.5 × 10^4^/cm^2^. The primary osteoblasts at passage 3–5 were utilized for further experiments. The protocols were approved by Ethics Review Board of Nanjing Medical University.

### RNA isolation and qRT-PCR

The protocols of quantitative real-time PCR (“qRT-PCR”) using the SYBR green kit and ABI-7600 FAST system (Applied Biosystems, Shanghai, China) were described previously [7, 10, 11]. The *mRNA primers* for *human TSC1* were described [46]. The *mRNA primers* for the human tuberin (*TSC2*) (catalog no. Hs00241068_s1) were purchased from Applied Biosystems (Shanghai, China). The *mRNA primers* of Nrf2-regulated genes, including *Nrf2, HO-1*, GCL-C and *NQO-1*, as well as *mRNA primers* of GAPDH were described [38, 39, 47, 48]. For analyzing miR, the miR was converted to cDNA using the First-Strand Synthesis Kit (SABiosciences, Frederick, MD). Human miR-19a (“-3p”) analysis was tested via the qRT-PCR assay using stem-loop primers, designed as previously described [49].

### Forced-expression of miR-19a

The lentiviral pSuper-GFP-puro-miR-19a expression vector (“LV-miR-19a”) was designed (based on the descried sequence [49]) and synthesized by Genepharm (Shanghai, China). The human osteoblasts were infected with LV-miR-19a lentivirus or the scramble non-sense control miRNA (“miRC”). Stable cells were selected by puromycin (0.5 μg/mL, Sigma). Expression of miR-19a (“-3p”) in the resulting cells was tested by qRT-PCR assay.

### TSC1 3′-UTR luciferase assay

*TSC1 mRNA’s* 3′-UTR was amplified via the primers as described [50], which was sub-cloned into pMIR-Report plasmid [50]. The reporter plasmid was then utilized as template to generate a miR-19a response element. The plasmid with the perfect match contain the complementary sequences of the mature miR-19a-3p behind the firefly luciferase gene [49]. OB-6 cells were transfected with luciferase reporter plasmid by Lipofectamine 2000. The luciferase activity was measured 24 hours after transfection using the Dual-Luciferase reporter assay system (Promega, Shanghai, China).

Cell survival, death and apoptosis assays. As described [7–11], cell survival, death and apoptosis were tested by the Cell Counting Kit-8 (CCK-8, Dojindo Laboratories, Kumamoto, Japan) assay, lactate dehydrogenase (LDH) release assay (Biyuntian, Wuxi, China) and the histone-DNA ELISA cell apoptosis plus kit (Roche, Shanghai, China), respectively [7–11].

### Western blotting assay

The detailed protocols for Western blotting assay were described in detail in our previous studies [6, 7, 11, 12]. The total gray of the protein band was quantified via ImageJ software, and the value was normalized to the loading control.

### shRNA

The lentiviral TSC1 shRNA (catalog no. sc-37437-V), the lentiviral Raptor shRNA (catalog no. sc-44069-V), and the lentiviral scramble control shRNA (catalog no.sc-108060) were all purchased from the Santa Cruz Biotech (Shanghai, China). The lentiviral shRNA (10 μL virus/1 mL medium, per well) was added directly to the cultured osteoblasts for 24 hours. Afterwards, puromycin (0.5 μg/mL, Sigma) was added to select stable cells. Knockdown of the targeted-protein in the stable cells was verified via Western blotting assay.

### Exogenous TSC1 expression

The full length human *TSC1 cDNA* was synthesized, sequence-verified and provided by Genepharm (Shanghai, China), which was inserted into the pSuper-puro-EGFP vector (Addgene, Shanghai, China). The construct was transfected to miR-19a-expressing osteoblasts via Lipofectamine 2000 transfection. Expression of TSC1 in the resulting cells was verified by the Western blotting assay.

### Reactive oxygen species (ROS) assay

As previously described [7–9, 11, 12], cellular ROS content was tested by the dichloro-dihydro-fluorescein diacetate (DCFH-DA) fluorescent dye (Invitrogen, Shanghai, China) assay.

### Statistical analysis

All values were expressed as means ± standard deviation (SD). The statistical significance of differences among groups were determined by one-way analysis of variance (ANOVA) followed by the Tukey's post hoc multiple comparison tests. *p* < 0.05 was considered significant.
